# Identifying Risk Groups in 73,000 Patients with Diabetes Receiving Total Hip Replacement: A Machine Learning Clustering Analysis

**DOI:** 10.3390/jpm15110537

**Published:** 2025-11-05

**Authors:** Alishah Ahmadi, Anthony J. Kaywood, Alejandra Chavarria, Oserekpamen Favour Omobhude, Adam Kiss, Mateusz Faltyn, Jason S. Hoellwarth

**Affiliations:** 1 School of Medicine, New York Medical College, Valhalla, NY 10595, USA; akaywood@student.nymc.edu (A.J.K.); achavarr@student.nymc.edu (A.C.); akiss@student.nymc.edu (A.K.); 2 Research & Development, Trilemma Capital, Vancouver, V6Z 2X7 BC, Canada; matt@trilemmacapital.com; 3 Department of Limb Lengthening and Complex Reconstruction, Hospital for Special Surgery, New York, NY 10021, USA; hoellwarthj@hss.edu

**Keywords:** total hip arthroplasty, diabetes mellitus, machine learning, clustering, non-routine discharge

## Abstract

**Background/Objective**: Diabetes mellitus (DM) is a highly prevalent condition that contributes to adverse outcomes in patients undergoing total hip arthroplasty (THA). This study applied machine learning clustering algorithms to identify comorbidity profiles among diabetic THA patients and evaluate their association with postoperative outcomes. **Methods**: The 2015–2021 National Inpatient Sample was queried using ICD-10 CM/PCS codes to identify DM patients undergoing THA. Forty-nine comorbidities, complications, and clinical covariates were incorporated into clustering analysis. The Davies–Bouldin and Calinski–Harabasz indices determined the optimal number of clusters. Multivariate logistic regression assessed risk of non-routine discharge (NRD), and Kruskal–Wallis H testing evaluated length-of-stay (LOS) differences. **Results**: A total of 73,606 patients were included. Six clusters were identified, ranging from 107 to 61,505 patients. Cluster 6, enriched for urinary tract infection and sepsis, had the highest risk of NRD (OR 7.83, *p* < 0.001) and the longest median LOS (9.0 days). Clusters 1–4 had shorter recoveries with median LOS of 2.0 days and narrow variability, while Cluster 5 showed intermediate outcomes. Kruskal–Wallis and post hoc testing confirmed significant differences across clusters (*p* < 0.001). **Conclusions**: Machine learning clustering of diabetic THA patients revealed six distinct groups with varied comorbidity profiles. Infection-driven clusters carried the highest risk for non-routine discharge and prolonged hospitalization. This approach provides a novel framework for risk stratification and may inform targeted perioperative management strategies.

## 1. Introduction

Diabetes mellitus (DM) is a group of metabolic diseases characterized by hyperglycemia due defects in insulin secretion, insulin action, or both [1]. In the United States, over 16 million individuals are affected by DM, a chronic condition with increasing prevalence and substantial public health implications [2]. Chronic, untreated DM exacerbates the risk of cardiovascular disease, stroke, retinopathy, and prolonged kidney damage [3]. Individuals with diabetes are also at an increased risk for postoperative complications such as infections, prolonged hospital stays, and higher mortality rates [4]. As such, DM significantly impacts post-operative outcomes for patients undergoing surgical procedures, including total hip arthroplasty (THA). THA provides reliable outcomes for patients suffering from end-stage degenerative hip osteoarthritis (OA), specifically pain relief, functional restoration, and overall improved quality of life [5]. Patients with type 2 diabetes undergoing revision hip and knee arthroplasty experience higher rates of in-hospital postoperative complications compared to non-diabetic patients [6]. Identifying underlying factors contributing to postoperative complications is essential for optimizing surgical care and improving patient outcomes.

While the general risks associated with diabetes in surgical contexts are well-documented, there is a growing interest in utilizing advanced analytical techniques to better understand and mitigate these risks for future patients [7]. Machine learning (ML) has been a promising tool for predicting patient outcomes and stratifying risks in various medical fields, including orthopedics. ML has shown to improve risk stratification by accurately predicting 30-day mortality following revision total hip and knee arthroplasty, outperforming the traditional methods of a CARDE-B score, 5, and 6-item Modified Frailty Index [8]. Recent work further emphasizes the growing use of machine learning models in orthopedic surgery, particularly in arthroplasty, where these tools are evaluated for outcome prediction, implant selection, and procedural planning [9,10]. Beyond orthopedics, ML-based tools are increasingly being validated in other areas of medicine, such as pharmacology, where multiple chatbots have been tested to benchmark their clinical reasoning capabilities [11], reflecting a broader integration of AI across healthcare disciplines. Similarly, clustering and optimization-based models have been used to enhance diabetes prediction accuracy, with Fuzz C-Means and Particle Swarm Optimization approaches demonstrating the growing role of unsupervised learning in modeling complex patient data [12]. The integration of ML algorithms into patient care enhances the quality and efficiency of healthcare delivery [13,14]. Previous unsupervised clustering methods of unstructured clinical notes and sequential EHR data have uncovered novel phenotypes and subgroups in heart failure and pediatric intensive care, with implications for personalized care and resource allocation [15].

Traditional predictive models utilize statistical analysis between two variables (i.e., linear regression, logistic regression, Pearson correlation coefficient, etc.). Clustering, on the other hand, is a statistical technique that organizes patients into groups (clusters) based on similarities across multiple variables [16]. In an unsupervised ML approach, an algorithm identifies patterns within a dataset without predefined categories or clinical endpoints [17]. This method can reveal hidden subgroups within heterogeneous patient populations, offering clinically meaningful risk profiles that may not be apparent through standard stratification methods [18]. Clustering does so by identifying patterns and naturally occurring subgroups within a dataset based on selected features, such as comorbidities, race, or gender. For example, most traditional studies of medical risk factors start by grouping patients by a comorbidity, such as DM or obesity, and group all these patients to assess the risk of an outcome, such as infection. ML does the reverse: it starts with the outcome of interest (infection) and evaluates a patient population sample to see which comorbidities end up having a similar level of occurrence (patients with DM and obesity may be clustered with patients who have urinary tract infection and also immunodeficiency because patients with these comorbidities have similar infection rates, despite their comorbidities not necessarily being the same).

This study applies machine learning-based clustering algorithms to a nationally representative cohort of patients with DM undergoing THA from the National Inpatient Sample (NIS) database. The primary aim is to determine the extent to which unsupervised clustering can stratify patients into distinct groups predictive of possible adverse outcomes, specifically non-routine discharge (NRD) and length of stay (LOS). While previous research has primarily relied on traditional regression-based approaches to identify individual risk factors, our study introduces an unsupervised, data-driven framework capable of uncovering latent comorbidity patterns that may better explain variability in outcomes among diabetic patients. This approach addresses a critical knowledge gap in preoperative risk assessment for diabetic patients, a population known to experience disparities in surgical outcomes [19]. Unlike prior clustering studies that primarily focused on disease prediction or general arthroplasty outcomes, this study applies ML to a national inpatient dataset to delineate comorbidity-based risk groups specific to diabetic patients undergoing THA, offering a novel framework for outcome stratification and preoperative optimization.

## 2. Materials and Methods

### 2.1. Data Source

This analysis was conducted utilizing data sourced from the 2015(Q4)-2021 National Inpatient Sample (NIS) Database, which was sponsored by the Agency for Healthcare Research and Quality (AHRQ) and developed for the Healthcare Cost and Utilization Project (HCUP). The NIS is the largest publicly available all-payer database on inpatient stays in the United States; its design approximates a 20% stratified sample of all admissions from long-term acute care hospitals and community hospitals.

### 2.2. Study Design and Population

This study is a retrospective clustering analysis of all diabetic individuals receiving Total Hip Arthroplasty. Hospitalizations for these patients were identified using diagnosis and procedure codes in the International Classification of Diseases Tenth Revision (ICD-10) Clinical Modification (ICD-10-CM) and Procedure Coding System (ICD-10-PCS). Hospitalizations including at least one of the procedure codes [‘0SR9019’, ‘0SR901A’, ‘0SR901Z’, ‘0SR9029’, ‘0SR902A’, ‘0SR902Z’, ‘0SR9039’, ‘0SR903A’, ‘0SR903Z’, ‘0SR9049’, ‘0SR904A’, ‘0SR904Z’, ‘0SR9069’, ‘0SR906A’, ‘0SR906Z’, ‘0SR907Z’, ‘0SR90EZ’, ‘0SR90J9’, ‘0SR90JA’, ‘0SR90JZ’, ‘0SR90KZ’, ‘0SRB019’, ‘0SRB01A’, ‘0SRB01Z’, ‘0SRB029’, ‘0SRB02A’, ‘0SRB02Z’, ‘0SRB039’, ‘0SRB03A’, ‘0SRB03Z’, ‘0SRB049’, ‘0SRB04A’, ‘0SRB04Z’, ‘0SRB069’, ‘0SRB06A’, ‘0SRB06Z’, ‘0SRB07Z’, ‘0SRB0EZ’, ‘0SRB0J9’, ‘0SRB0JA’, ‘0SRB0JZ’, ‘0SRB0KZ’] between 2015 Q4 and 2019 were included in the study population. Entries missing data in the age, sex, race, income quartile or payer type columns were excluded from this analysis.

### 2.3. Data Variables and Outcomes

Demographic variables included in this analysis were age, sex, race (White, Black, Hispanic, Asian or Pacific Islander, Native American, Other), household income quartile based on patient’s ZIP code, and the expected primary payer type (Medicare, Medicaid, Private insurance, Self-pay, No Charge, Other).

Variables utilized for clustering were a total of 49 clinical comorbidities, complications and in-hospital covariates, the diagnosis, procedure and Elixhauser Comorbidity column (CMR_xxx) that was used to define each clustering variable is included in Supplementary Table S1, the full list of variables includes the following:

Alcohol Abuse, Autoimmune Disease, Dementia, Drug Abuse, Obesity, Peripheral Vascular Disease, Hypertension, Hyperlipidemia, Cancer, Myocardial Infarction, Heart Failure, Acute Kidney Failure, Chronic Kidney Disease, Pulmonary Embolism, Chronic Obstructive Pulmonary Disease (COPD), Smoking, Deep Vein Thrombosis (DVT), Chronic Liver Disease, Depression, Anxiety, HIV/AIDS, Dependent Status, Acute Ischemic Stroke, Anemia, Coagulopathy, Hypothyroid, Hyperthyroid, Arrhythmia, Sleep Apnea, Inflammatory Bowel Disease (IBD), Osteoporosis, Hypoxic/Anoxic CNS & PNS Damage, Postprocedural CSF Leak, Intraprocedural Dural Tear, Post/Intraprocedural Hemorrhage/Hematoma, Wound Dehiscence, Sepsis, Pneumonia, Postprocedural Urinary Tract Infection, Acute Respiratory Distress Syndrome (ARDS), Aspiration Pneumonitis, Dysphagia, Seizures and Status Epilepticus, Hydrocephalus, Cerebral Edema, Cerebral Herniation, Tracheostomy, EVD Placement.

Outcomes analyzed were non-routine discharge (NRD) and length of stay (LOS). NRD was defined by values other than 1 in the column indicating patient disposition “DISPUNIFORM”, where a value of 1 indicates a routine discharge. Length of stay was a continuous variable extracted from the “LOS” column of the NIS database.

### 2.4. Clustering

To identify distinct profiles of comorbidities and covariates among patients, clustering was performed using the k-modes unsupervised machine learning algorithm. K-modes was selected as it is computationally efficient and designed for handling categorical data that is both highly dimensional and highly sparse as seen in the available NIS data on comorbidities and clinical course variables. The K-modes algorithm’s partitioning of clusters based on attribute mismatches makes it preferable over probabilistic models that experience degrading performance with high counts of sparse variables. Minimal data assumptions in k-modes also enable purely data-driven discovery of patterns within large datasets. Cao initialization was selected over random or huang initialization as density-based initialization was more consistent with the objective of identifying subgroups in the patient sample.

**Figure 1:**
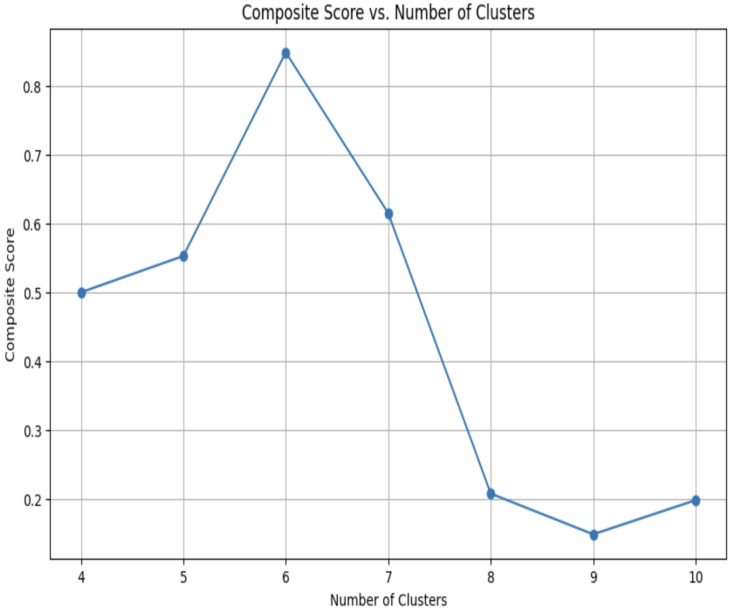
Composite DBI-CHI Scoring for Determination of Optimal Number of Clusters.

The optimal number of clusters was determined to be six using a composite Davies–Bouldin Index (DBI) and Calinski–Harabasz Index (CHI), where an inverted and normalized DBI score is used in conjunction with a normalized CHI score. Figure 1 displays the composite DBI-CHI scores plotted against the number of clusters ranging from 4 to 10 which the model was fitted to. A minimum of 4 clusters was chosen as few clusters (2–3) often force data into overly broad groupings or capture only the most dominant feature variation. The curve demonstrates a peak at six clusters, indicating the most favorable clustering configuration based on internal validity metrics. The composite score increases from 0.50 at four clusters to a maximum of 0.85 at six clusters, after which it declines sharply.

### 2.5. Statistical Analysis

Descriptive statistics for demographic characteristics of the entire sample as well as individual clusters were calculated to observe trends.

Clusters were displayed in a heatmap specifying the mean prevalence of each clustering variable within the given cluster. Demographic variables of each cluster were recorded for observation of trends. Where possible, Fisher’s Exact Test and Chi-Square Tests were used in pairwise comparisons of comorbidities/covariates between clusters to assess significant differences in comorbidity prevalence between clusters.

Multivariable logistic regression was performed to calculate odds ratios and 95% confidence intervals (95% CIs) of the adjusted odds of NRD of all clusters relative to Cluster 1. Age, sex, race, income quartile and payer type were controlled for. Unadjusted results for each regression are also displayed.

**Figure 2:**
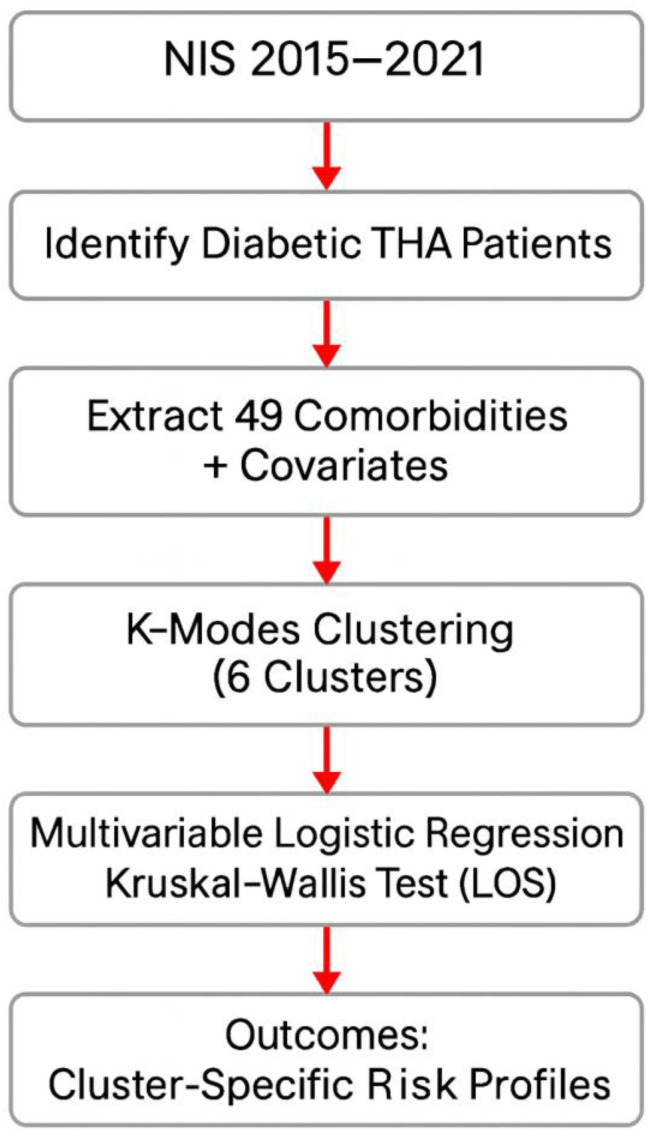
Methodological pipeline illustrating data extraction, clustering, and outcome analysis.

Given that length of stay is a continuous variable that may not follow a normal distribution, Kruskal–Wallis H-Testing was performed to assess significant differences in median length of stay between clusters. This was followed by post hoc pairwise comparison between clusters with Bonferroni correction to identify specific cluster differences. Length-of-stay distributions were visualized through box and whiskers plot, violin plot and density plot. Descriptive statistics including the median, first quartile (Q1), third quartile (Q3), and interquartile ranges were also calculated to further characterize the distribution of length of stay across clusters. All analyses were performed using Python version 3.12.7 leveraging the pandas, matplotlib, k-modes, pyarrow, seaborn, and scikit-learn packages. Figure 2 demonstrates a metholodolgical pipeline sequence used in the analysis of the dataset. 

## 3. Results

A total of 73,606 patients with diabetes mellitus (DM) who underwent total hip arthroplasty (THA) were included in the study. The mean age of the cohort was 68.1 years with a standard deviation of 10.0 years. The sex distribution was 51.36% identifying as female (*n* = 37,806) and 48.64% as male (*n* = 35,800).

As shown in Table 1, patients were distributed across income quartiles as follows: 24.56% (*n* = 18,079) in the first quartile (lowest income), 27.17% (*n* = 20,001) in the second quartile, 26.26% (*n* = 19,326) in the third quartile, and 22.01% (*n* = 16,200) in the fourth quartile (highest income). Most patients were covered by Medicare (65.04%, *n* = 47,870), followed by Medicaid (26.70%, *n* = 19,656). Private insurance accounted for 4.93% (*n* = 3628) of the sample, while 2.62% (*n* = 1931) of patients were self-pay. A smaller proportion of patients were classified under no charge (0.65%, *n* = 477) or other (0.06%, *n* = 44) payment categories.

Racial demographics revealed most the cohort as white (79.96%, *n* = 58,854). Black patients comprised 11.63% (*n* = 8559) of the sample, while Hispanic patients made up 4.86% (*n* = 3575). Asian or Pacific Islander patients accounted for 1.27% (*n* = 935), Native American patients for 0.46% (*n* = 340), and 1.82% (*n* = 1343) identified as Other.

### 3.1. Clustering Comorbidities

**Figure 3:**
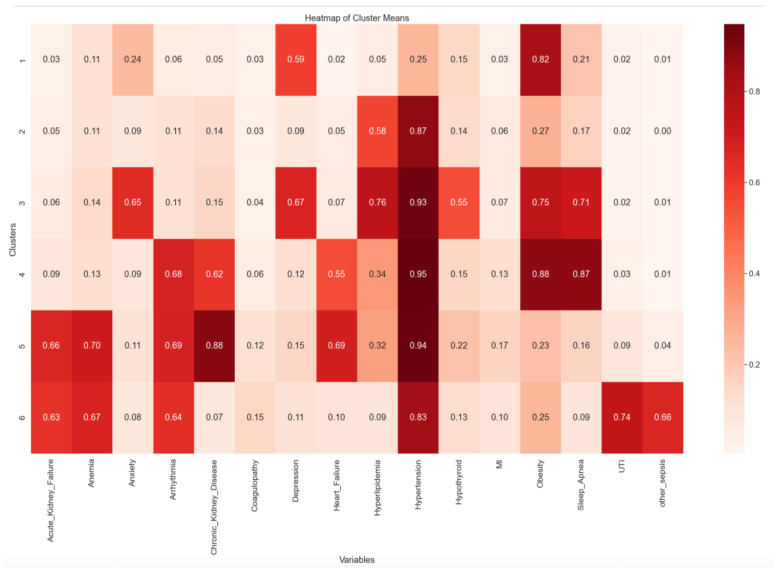
Heatmap indicating mean prevalence of each comorbidity/covariate within clusters 1–6; Clusters are numbered by increasing rates of non-routine discharge (NRD).

By selection criteria, all patients have diabetes mellitus. The six clusters are differentiated by an increasing rate of NRD and longer LOS, with Cluster 6 having the highest rate of NRD and longest LOS. Among the comorbidity profiles demonstrated in Figure 3, Cluster 1 and Cluster 2 were primarily defined by elevated rates of obesity and hypertension, respectively. Cluster 3 demonstrated the highest prevalence of depression and hyperlipidemia. Cluster 4 was characterized by increased rates of obesity and sleep apnea. Cluster 5 was defined by the highest prevalence of chronic kidney disease and heart failure, suggesting greater cardiometabolic burden. Cluster 6 displayed the highest rates of urinary tract infections (UTIs) and sepsis, indicating a clinically vulnerable subgroup with potentially more severe systemic health risks, which lead to higher rates of NRD and LOS.

### 3.2. Non-Routine Discharge

The primary aim of this study was to assess the extent ML can predict adverse events within the NIS database for diabetic patients undergoing THA. The first outcome of the study was NRD. Table 2 presents the results of multivariable logistic regression assessing the association between cluster membership and NRD, using Cluster 1 (*n* = 3372) as the standard reference of comparison for risk. Cluster 2 (*n* = 61,505) demonstrated a slightly elevated unadjusted odds of NRD (OR: 1.10; 95% CI: 1.02–1.18; *p* = 0.010), though this association was not significant after adjustment (aOR: 0.94; 95% CI: 0.87–1.02; *p* = 0.128). Similarly, Cluster 3 (*n* = 5174) had a higher crude OR (1.16; 95% CI: 1.06–1.27; *p* = 0.002), but the adjusted estimate was non-significant (aOR: 1.06; 95% CI: 0.96–1.16; *p* = 0.261). In contrast, Cluster 4 (*n* = 1916) maintained a significantly increased risk both before (OR: 1.51; 95% CI: 1.33–1.71) and after adjustment (aOR: 1.33; 95% CI: 1.16–1.53; both *p* < 0.001). Notably, Clusters 5 (*n* = 1532) and 6 (*n* = 107) demonstrated the highest risk for NRD. Cluster 5 exhibited an aOR of 3.18 (95% CI: 2.62–3.87; *p* < 0.001), while Cluster 6 had the highest aOR of 7.83 (95% CI: 3.16–19.41; *p* < 0.001), indicating substantially elevated odds of NRD even after adjusting for age, sex, race, income quartile, and payer type.

### 3.3. Length of Stay

**Figure 4:**
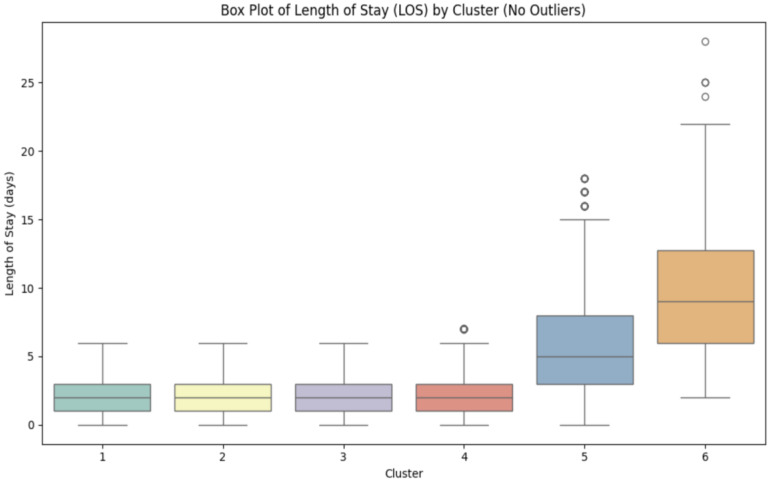
Box-plot comparing the distribution of length of stay (LOS) across all six cluster groups.

Another outcome of the study was assessing the length of stay (LOS) within each cluster group. As shown in Figure 4 and detailed in Table 3, Clusters 1 through 4 had identical median postoperative hospitalizations of 2.0 days, with narrow interquartile ranges (IQR = 2.0), suggesting shorter recovery periods across these groups. In contrast, cluster 5 and 6 patients experienced significantly higher LOS. Cluster 5 demonstrated a median LOS of 5.0 days (IQR = 5.0), while Cluster 6 exhibited the most prolonged hospital stay with a median LOS of 9.0 days with a wide interquartile range (IQR = 6.75).

## 4. Discussion

In this study, we utilized an unsupervised machine learning algorithm (k-modes clustering algorithm) to separate diabetic patients undergoing total hip arthroplasty (THA) into clinically relevant subgroups based on distinct comorbidity profiles. We aimed to analyze this population for subgroups with increased risk of non-routine discharge and length of stay, based on the multidimensional intersection of clinical comorbidities and features of their course of hospitalization. Using the NIS database, a cohort of 73,606 patients with diabetes mellitus undergoing THA was identified. This cohort was algorithmically separated into clusters based on their similarity across 49 distinct comorbidities and clinical course covariates. Six unique patient clusters were identified within this cohort, each demonstrating significant differences in non-routine discharge rates and length of stay (LOS). These outcomes reflect hospital resource utilization more than direct measures of patient morbidity or mortality, and should be interpreted as indicators of care complexity and demand for resources rather than as adverse clinical outcomes. For example, a longer LOS or non-routine discharge should not be assumed to reflect poor recovery; it may instead signify appropriate, high-quality care delivered to meet the needs of medically complex patients. Cluster 6 was linked to the longest length of stay and the highest prevalence of urinary tract infections (UTI) and sepsis, suggesting that infection may substantially increases inpatient resource utilization. However, Cluster 6 consisted of a relatively small subgroup (*n* = 107), and therefore these findings should be interpreted with caution due to reduced statistical stability. Alternatively, both clusters 1 and 3 exhibited lower rates of non-routine discharge and shorter hospital stays, indicating a relatively stable postoperative period within this diabetic subpopulation. These results show the value of unsupervised clustering for finding latent subgroups that impact perioperative resource use, providing a framework for identifying risk groups rather than direct prediction of clinical outcomes.

The cluster profiles identified in our study align with previously described high-risk subgroups in both orthopedic and general surgical literature. Diabetes mellitus (DM), along with renal disease, peripheral vascular disease (PVD), and chronic pulmonary disease (COPD), are established independent predictors of prolonged length of stay (LOS) following total hip arthroplasty (THA) [20]. The presence of multiple comorbidities compounds this effect, further increasing postoperative risk. In addition, machine learning models have been shown to consistently outperform traditional risk predictors (linear regression, numerical analysis, etc.) for complications, readmissions, and functional outcomes following total joint arthroplasty [21]. Rather than replacing these models, unsupervised clustering can be used as a complementary method for recognizing unique patient profiles that may better explain variability in perioperative outcomes.

These results support the growing use of machine learning in clinical research. Although the clusters help define patient subgroups, their clinical relevance must be confirmed through interpretation and prospective validation. Meaningful adoption of ML in arthroplasty will rely on producing outputs that are not only accurate, but clinically interpretable and actionable [21].

Cluster 2 initially appeared to have a higher rate of non-routine discharge (unadjusted odds ratio of 1.10, 95% CI: 1.02–1.18; *p* = 0.010). However, after adjusting for payer and demographic variables, the association was no longer significant (adjusted OR 0.94, 95% CI: 0.87–1.02; *p* = 0.128), suggesting that the difference was largely attributed to socioeconomic factors. This finding is consistent with mounting evidence for the association of social determinants of health (SDOH) with outcomes of orthopedic and general surgery [22]. Furthermore, Cluster 6′s high rate of infections and longer LOS in the absence of worsened disposition outcomes may reflect the efficacy of inpatient management regimens in preventing adverse events at the expense of significant resource use. Future research should determine whether early cluster identification can help guide usage of postoperative resources rather than make direct links to clinical morbidity.

Several study limitations must be emphasized. Although our clustering analysis identifies meaningful subgroups, the causative mechanisms underlying these patterns cannot be determined. Our study design does not permit causal inference, and uncontrolled confounding variables can potentially be responsible for the observed outcomes. Furthermore, while non-routine discharge and LOS are often used in health services as proxies for complexity or burden of care, they are not always predictive of adverse clinical outcomes from a patient perspective. Patient LOS can be longer, or they may be discharged (NRD) to a facility while still having optimal pain control, mobility and surgical results. These outcomes are instead more direct measures of hospital resource allocation rather than recovery quality. For instance, comorbid conditions such as cardiovascular disease, renal failure, and poor glycemic control are known to increase postoperative complications in diabetic surgical patients; however, the extent of their influence could not be fully captured in this study due to data constraints [20,23]. The NIS database lacks key clinical variables such as preoperative hemoglobin A1c, kidney or liver function, or body mass index, all of which have known correlations with outcomes following arthroplasty [23,24,25]. Importantly, the dataset does not contain intraoperative information such as operative time, implant type, or anesthetic modality, and it does not allow for longitudinal tracking of the same patient beyond discharge. Collectively, these limitations restrict the precision of our analyses and limit generalizability of findings.

Future research could benefit from integration with more clinically detailed datasets. Linking to electronic health records (EHRs) or national joint replacement registries would make it possible to incorporate vital signs, laboratory values, imaging studies, medication regimens, and operative details. Additionally, the smallest subgroup identified in this study (Cluster 6) included a limited sample size (*n* = 107), and therefore its findings should be interpreted as observational rather than definitive. Larger, prospectively collected datasets are needed to validate the consistency of this cluster and confirm whether the observed trends persist. Integration of genomic, metabolic, and inflammatory biomarkers could also help clarify mechanisms underlying unique risk phenotypes. Incorporating such data will improve cluster resolution and provide new insights into biological processes underlying postoperative outcome. Prospective validation across institutions will be essential to assess reproducibility and evaluate whether targeted perioperative interventions can reduce adverse outcomes among high-risk clusters. These efforts would allow for more precise and evidence-based application of machine learning clustering to orthopedic care.

## 5. Conclusions

This study employed an unsupervised machine learning clustering approach to stratify a nationally representative cohort of patients with diabetes mellitus undergoing total hip arthroplasty, identifying six clinically distinct subgroups with differing comorbidity profiles and varying levels of hospital resource utilization. Cluster 6, characterized by higher rates of urinary tract infections and sepsis, exhibited the longest hospitalization and greatest resource demand, while clusters 1 and 3 demonstrated shorter recovery time. These findings emphasize the heterogeneity within the diabetic population and show how unsupervised clustering can uncover patterns of comorbidity associated with different patient outcomes. However, the results should be interpreted with caution rather than used for direct clinical decision making. Because the analysis relies on administrative data without perioperative variables or follow-up, further research is needed to reveal if clusters correspond to meaningful differences in patient outcomes. Prospective validation, clinical and laboratory data, and postoperative follow-up are necessary to see whether clustering can be used to optimize care planning. This work is presently a data-driven foundation for future efforts to refine risk stratification amongst diabetic patients undergoing total hip arthroplasty.

## Figures and Tables

**Table 1:** Demographics of patients undergoing total hip arthroplasty (THA) with diabetes.

Sample Size		Age (Years)		Sex	*n* (%)
Total	73,606	Mean	68.12	Male	35,800 (48.64)
		St. Dev.	10.003	Female	37,806 (51.36)
Income Quartile	*n* (%)	Payer Type	*n* (%)	Race	*n* (%)
1st	18,079 (24.56)	Medicare	47,870 (65.04)	White	58,854 (79.96)
2nd	20,001 (27.17)	Medicaid	19,656 (26.70)	Black	8559 (11.63)
3rd	19,326 (26.26)	Private insurance	3628 (4.93)	Hispanic	3575 (4.86)
4th	16,200 (22.01)	Self-pay	1931 (2.62)	Asian or Pacific Islander	935 (1.27)
		No charge	477 (0.65)	Native American	340 (0.46)
		Other	44 (0.06)	Other	1343 (1.82)

**Table 2:** Multivariable Logistic Regression of Non-Routine Discharge with Cluster 1 as the reference group. Age, sex, race, income quartile and payer type are controlled for in the aORs.

Cluster	Cluster Size	Odds Ratio (95% CI)	*p*-Value	Adjusted Odds Ratio (95% CI)	*p*-Value
1	3372	Ref.	Ref.	Ref.	Ref.
2	61,505	1.10 (1.02–1.18)	0.010	0.94 (0.87–1.02)	0.128
3	5174	1.16 (1.06–1.27)	0.002	1.06 (0.96–1.16)	0.261
4	1916	1.51 (1.33–1.71)	<0.001	1.33 (1.16–1.53)	<0.001
5	1532	4.33 (3.63–5.16)	<0.001	3.18 (2.62–3.87)	<0.001
6	107	10.88 (4.42–26.78)	<0.001	7.83 (3.16–19.41)	<0.001

**Table 3:** Median number of days, first quartile, third quartile, and interquartile ranges for each cluster group.

Cluster Group	Median Days Spent Hospitalized Post-Surgery	Q1	Q3	IQR
1	2.00	1.00	3.00	2.00
2	2.00	1.00	3.00	2.00
3	2.00	1.00	3.00	2.00
4	2.00	1.00	3.00	2.00
5	5.00	3.00	8.00	5.00
6	9.00	6.00	12.75	6.75

## Data Availability

The original contributions presented in this study are included in the article. Further inquiries can be directed to the corresponding author.

## Supplementary Materials

The following supporting information can be downloaded at: https://www.mdpi.com/article/10.3390/jpm15110537/s1, Figure S1. Heatmap indicating mean prevalence of all comorbidity/covariate within clusters 1–6; Clusters are numbered by increasing rates of non-routine discharge; Table S1. Inclusion criteria and all variables along with ICD-10 codes analyzed for each cluster group.
